# Comparison of modified one-layer and formal two-layer microsurgical vasovasostomy for vasectomy reversal

**DOI:** 10.1186/s12610-026-00323-3

**Published:** 2026-08-04

**Authors:** Junlong Wang, Junhui Wu, Jiali Li, Wenhao Zhang, Dengxiong Li, Jiasheng Yan, Xiaodong Jin

**Affiliations:** Department of Urology, The First Affiliated Hospital of Zhejiang Chinese Medical University (Zhejiang Provincial Hospital of Chinese Medicine), Hangzhou, 310006 China

**Keywords:** Vas deferens obstruction, Vasectomy, Vasectomy reversal, Vasovasostomy, Microsurgery, Obstruction du Canal déférent, Vasectomie, Inversion de la Vasectomie, Vaso-Vasostomie, Microchirurgie

## Abstract

**Background:**

The formal two-layer microsurgical technique is considered the gold standard for vasovasostomy (VV) because of its excellent outcomes; however, it is technically demanding and time-consuming. In this study, we compared the outcomes of modified one-layer and formal two-layer microsurgical VV in men undergoing VV.

**Results:**

This retrospective analysis included 62 men who underwent bilateral microsurgical VV performed by a single surgeon between 2017 and 2024. The formal two-layer and modified one-layer techniques were performed in 27 and 35 men, respectively. The mean operative time was significantly shorter with the modified one-layer technique than with the formal two-layer technique (118.89 ± 7.99 vs. 152.52 ± 9.31 min, *P* < 0.001). Patency rates (91.43% [32/35] vs. 92.59% [25/27], *P* = 0.448) and pregnancy rates (34.29% [12/35] vs. 37.04% [10/27], *P* = 0.822) were comparable between the two groups. Postoperative sperm concentration and motility did not differ between the two groups. No complications and postoperative reobstruction were observed in either group.

**Conclusions:**

Compared with formal two-layer microsurgical VV, the modified one-layer technique achieved comparable patency and pregnancy rates while reducing technical difficulty and operative time. It may therefore represent an alternative surgical approach with potential clinical benefits for VV.

## Résumé

**Contexte:**

La technique microchirurgicale officielle à deux couches est considérée comme la référence absolue pour la vaso-vasostomie (VV) en raison de ses excellents résultats ; cependant, elle est techniquement exigeante et chronophage. Dans cette étude, nous avons comparé les résultats de la VV microchirurgicale modifiée à une couche et celle officielle à deux couches, chez des hommes en demande de VV.

**Résultats:**

Cette analyse rétrospective a inclus 62 hommes bénéficiant d’une VV microchirurgicale bilatérale réalisée par un seul chirurgien entre 2017 et 2024. Les techniques officielle à deux couches et à une couche modifiée ont été réalisées respectivement avec 27 et 35 hommes. Le temps opératoire moyen était nettement plus court avec la technique modifiée à une couche qu’avec la technique officielle à deux couches (118,89 ± 7,99 versus 152,52 ± 9,31 min, *p* < 0,001). Les taux de perméabilisation (91,4 % [32/35] versus 92,6 % [25/27] *p* = 0,448) et les taux de grossesse (34,3 % [35/12] versus 37,0 % [27/10] *p* = 0,822) étaient comparables entre les deux groupes. La concentration et la mobilité postopératoires des spermatozoïdes ne différaient pas entre les deux groupes. Aucune complication ni ré-obstruction postopératoires n’ont été observées dans aucun des deux groupes.

**Conclusion:**

Comparée à la VV microchirurgicale officielle à deux couches, la technique modifiée à une couche a permis d’atteindre des taux de perméabilité et de grossesse comparables, tout en réduisant la difficulté technique et le temps opératoire. Par conséquent, cela pourrait représenter une approche chirurgicale alternative avec des bénéfices cliniques potentiels pour la VV.

## Introduction

Vasectomy, performed for contraceptive purposes, is the most common cause of vas deferens obstruction. It has been widely adopted worldwide and represents one of the main methods of contraception. Approximately 6% to 8% of couples worldwide choose vasectomy as their method of contraception [1]. However, approximately 6% to 10% of men who have undergone vasectomy subsequently seek vasectomy reversal to restore fertility for reasons such as remarriage, a desire for additional children, or child bereavement [2, 3]. In the United States alone, where approximately 500,000 vasectomies are performed annually, roughly 6% of men ultimately undergo vasectomy reversal for various reasons [4–6]. In recent years, changes in China’s family planning policy have allowed numerous families to have additional offspring, resulting in a significant increase in the demand for vasectomy reversal in China [7, 8]. Vasectomy reversal is the only approach that enables natural conception and offers the potential for multiple future pregnancies, while also being more cost-effective than assisted reproductive technology (ART) [9]. Notably, only 15% of men undergoing vasectomy reversal require vasoepididymostomy (VE); consequently, vasovasostomy (VV) accounts for the majority of these procedures and remains the most common treatment for vas deferens obstruction [10, 11].

Microsurgical VV has emerged as the preferred method of vasectomy reversal [12, 13]. Microsurgical VV primarily encompasses one-layer and two-layer technique. The two-layer technique, which involves suturing the mucosal layer followed by the seromuscular layer, provides better access to the lumen and superior tightness. However, this technique is relatively complex, resulting in a longer operative time. In contrast, the one-layer technique utilizes full-thickness sutures, which is technically simpler and reduces operative time; however, it may compromise the watertight closure [11, 14, 15]. Most microsurgeons favor the two-layer technique [11], of which the formal two-layer microsurgical microdot technique—first introduced by Goldstein—has become the gold standard for VV [16, 17]. Before the anastomosis, the two ends of the vas deferens are marked with a micromarker pen to ensure optimal suture placement and achieve a watertight anastomosis [15]. However, the human vas deferens has an internal diameter of only 300–400 μm; consequently, placing 6–8 sutures in the mucosal layer poses substantial technical challenges, including difficulties with precise needle placement and suture handling. The complexity of the procedure requires exceptional high microsurgical skills, resulting in a steep learning curve, prolonged operative time, and limited clinical application [1, 18, 19].

Our team introduced a modified one-layer microsurgical VV technique that incorporates elements of the microdot technique and adds seromuscular sutures in addition to full-thickness sutures. This approach combines the advantages of the formal two-layer and conventional one-layer technique, yielding promising results. In this retrospective comparative study, we evaluated the efficacy of this novel technique compared with that of formal two-layer VV. Our findings provides an alternative surgical approach with potential clinical benefits for VV.

## Participants and methods

### Study design and patients

**Fig. 1:**
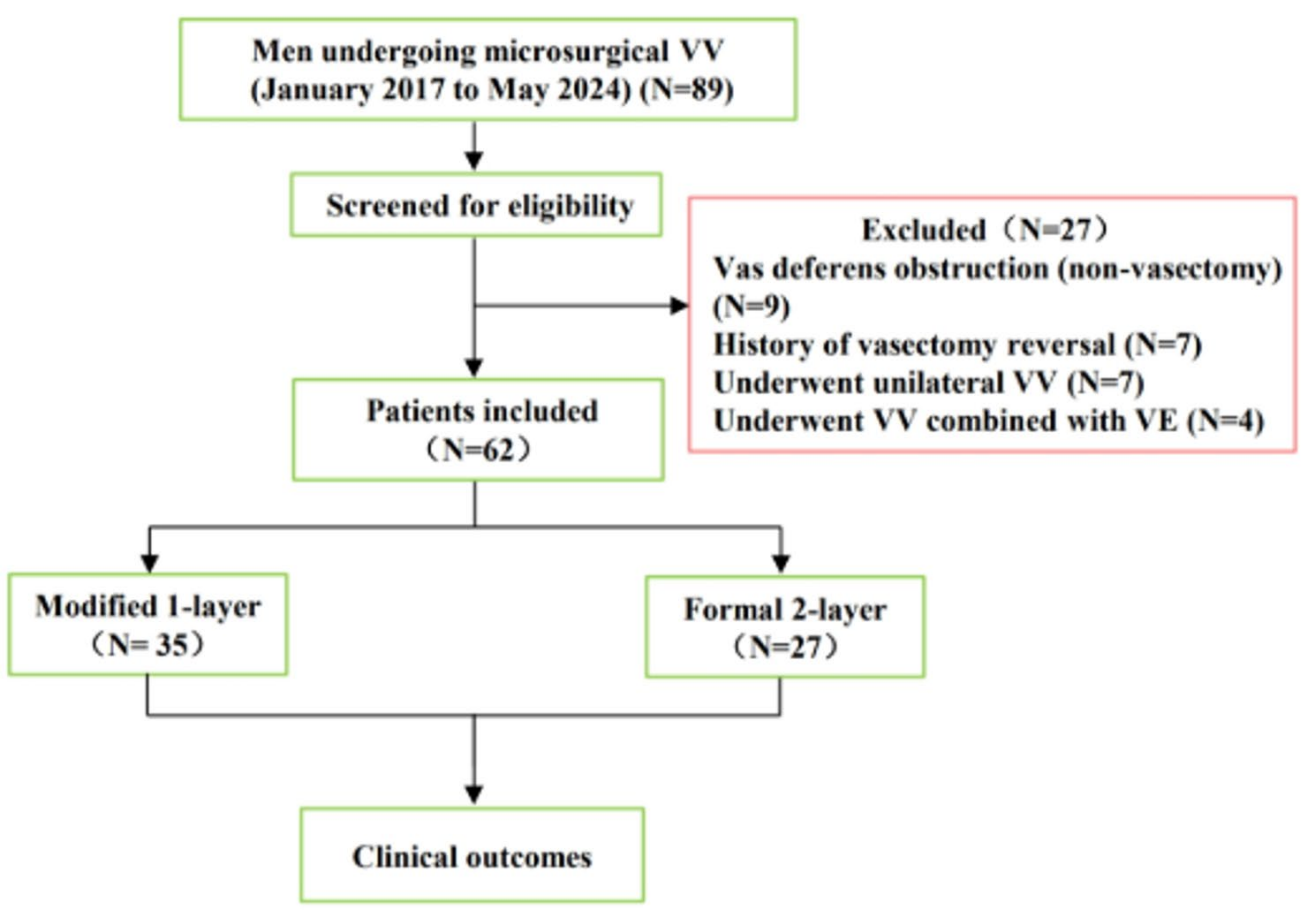
Study Flowchart Illustrating Patient Inclusion and Exclusion. Abbreviations: VE, vasoepididymostomy; VV, vasovasostomy

This study retrospectively analyzed men who underwent microsurgical VV performed by a single surgeon experienced in microsurgery (Jin XD) between January 2017 and May 2024. All men had previously undergone vasectomy and sought vasectomy reversal to restore fertility. The inclusion criteria were as follows: (1) primary VV after vasectomy; (2) absence of sperm in at least two consecutive semen analyses; (3) follicle-stimulating hormone, luteinizing hormone, and testosterone levels within the normal ranges; (4) normal testicular volume and a dilated proximal vas deferens observed on ultrasonography; and (5) completion of bilateral VV during surgery. Men were excluded if they underwent VE or VV for causes other than prior vasectomy, such as epididymitis, herniorrhaphy, or orchidopexy. In addition, men who underwent unilateral VV only or had a history of vasectomy reversal were also excluded. In a pilot study, an initial cohort of five men underwent the modified one-layer VV. Given the acceptable outcomes observed during this pilot phase, we subsequently performed this technique in additional patients. From January 2017 to May 2024, a total of 89 men with vas deferens obstruction underwent either formal two-layer VV or the modified one-layer VV in a randomized manner. The final cohort comprised 62 men, of whom 27 men underwent the formal two-layer technique and 35 men underwent the modified one-layer technique. The participant flow diagram is presented in Fig. 1.

The study was approved by the local ethics committee. Given the retrospective nature of the study, the committee has waived the requirement for individual informed consent.

### Surgical techniques

**Fig. 2:**
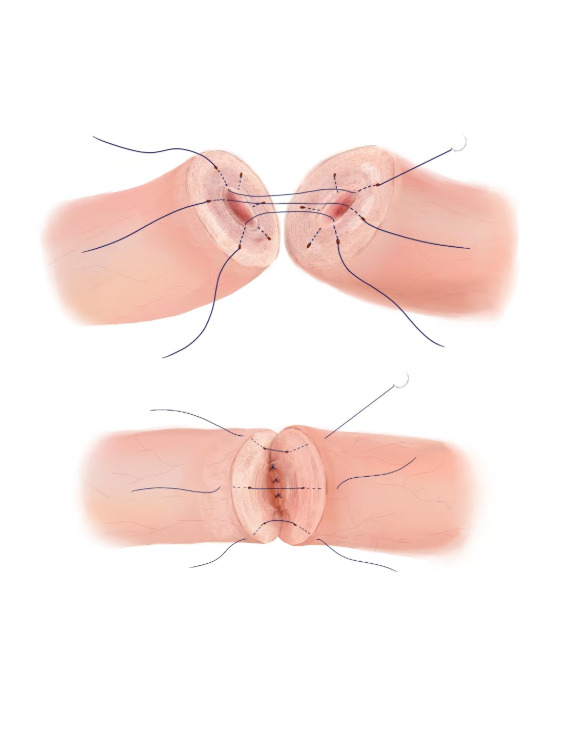
Formal two-layer vasovasostomy. Six interrupted sutures were used to reapproximate the mucosal layer of the vas deferens, followed by six interrupted sutures to reapproximate the seromuscular layer

**Fig. 3:**
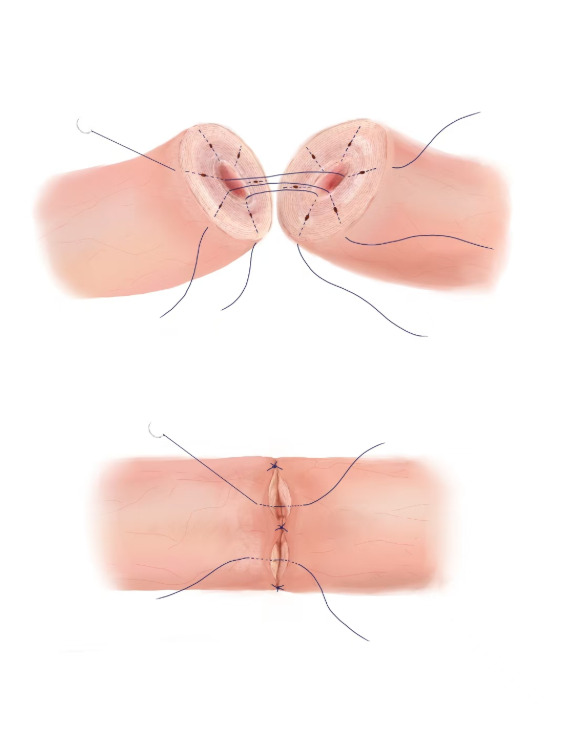
Modified one-layer vasovasostomy. Six full-thickness sutures were used to reapproximate both the muscular and mucosal layers of the vas deferens, followed by six interrupted sutures to reapproximate the intervening seromuscular layer

Following general anesthesia, a midline scrotal incision was made to expose the testis. The two ends of the vasectomy site were identified and isolated 2.0 cm both proximal and distal to the vasectomy site. The scar tissue at both ends was excised vertically. After confirming patency of the distal end, vasal fluid obtained from the proximal vas deferens was collected for microscopic examination. If sperm or sperm fragments were identified, microsurgical VV would be performed. The ends of the vas deferens were approximated using a Goldstein Microspike approximator clamp. Under a surgical microscope (Leica, Germany), a microtip marking pen was used to mark the planned needle entry or exit points at the 12, 2, 4, 6, 8, and 10 o’clock positions between the mucosal and serosal layers on both cross-sections of the vas deferens. Sutures were subsequently placed at the marked microdots. Formal two-layer technique (as described by Goldstein [15]): the mucosal layer of the vas deferens was anastomosed using six interrupted 10 − 0 polypropylene sutures, and the seromuscular layer was then approximated with six interrupted 9 − 0 polypropylene sutures placed midway between the mucosal sutures (Fig. 2). Modified one-layer technique: the vas deferens was anastomosed with six full-thickness 9 − 0 polypropylene sutures to reapproximate both the muscular and mucosal layers; subsequently, the seromuscular layer was reinforced with six additional interrupted 9 − 0 polypropylene sutures positioned midway between the full-thickness sutures (Fig. 3).

All patients underwent routine follow-up assessments. Semen analysis was performed at 6 weeks postoperatively and then every 3 months thereafter throughout the 2-year follow-up period or until pregnancy was achieved. Data on operative time, postoperative semen analysis results, complications, postoperative reobstruction, and pregnancy were recorded. Patency was defined as the presence of motile sperm in the ejaculate during follow-up evaluations. Pregnancy was defined as spontaneous conception and was confirmed by the presence of fetal cardiac activity at 6–7 weeks of gestation.

### Statistical methods

SPSS software (version 26) was used for statistical analysis. The modified one-layer and formal two-layer technique groups were compared in terms of operative efficiency and postoperative outcomes. Continuous variables were expressed as the mean ± standard deviation or median (range), depending on the data distribution, and were compared using the Student’s t-test or the Mann–Whitney U test, as appropriate. Categorical variables, including patency, pregnancy, and complication rates, were compared using the chi-square test or Fisher’s exact test. All statistical tests were two-sided, and *P* < 0.05 was considered statistically significant.

## Results

### Clinical characteristics of the included patients

The patient characteristics are presented in the Table 1. Patient age and the presence of sperm granuloma were similar between the two groups. The median obstructive interval was 7.29 years (range, 2–20 years) in the modified one-layer group and 6.85 years (range, 4–11 years) in the formal two-layer group (*P* = 0.54).

**Table 1:** Characteristics of patients undergoing vasovasostomy

	Modified 1-layer(*n*=35)	Formal 2-layer(*n*=27)	*P* value
Age (years)	39.69 ± 5.18	40.22 ± 2.88	0.631
Obstructive interval(years)	7.29 ± 3.23	6.85 ± 1.96	0.54
Sperm granulomapresence (%)	31.43% (11/35)	33.33% (9/27)	0.871

Data are presented as the mean ± standard deviation for normally distributed continuous variables and as numbers (percentages) for categorical variables. Statistical comparisons were performed using the independent-samples t-test for normally distributed continuous variables and the chi-square test or Fisher’s exact test for categorical variables, as appropriate

### Operative time and postoperative outcomes

The modified one-layer technique significantly shortened the operative time compared with the formal two-layer technique (118.89 ± 7.99 vs. 152.52 ± 9.31 min, *P* < 0.001) (Table 2). The modified one-layer and formal two-layer groups had comparable follow-up durations (mean, 20.03 [6–24] vs. 19.48 [5–24] months) and mean times to patency (3.86 [1.5–18] vs. 4.02 [1.5–16] months). Patency rates (91.43% [32/35] vs. 92.59% [25/27], *P* = 0.448) and pregnancy rates (34.29% [12/35] vs. 37.04% [10/27], *P* = 0.822) were similar between the two groups. Postoperative sperm concentration and motility did not differ significantly between the two groups, and no complications or postoperative reobstruction occurred in either group.

**Table 2:** Outcomes of modified one-layer vasovasostomy compared with formal two-layer vasovasostomy

	Modified 1-layer (*n* = 35)	Formal 2-layer (*n* = 27)	*P* value
Operation time (minutes)	118.89 ± 7.99	152.52 ± 9.31	< 0.001
Patency (%)	91.43% (32/35)	92.59% (25/27)	0.448
Sperm concentration (×10^6^/ml)	29.13 ± 12.15	30.72 ± 12.44	0.614
Sperm motility (%)	35.78 ± 13.59	35.07 ± 12.86	0.837
Pregnancy rate (%)	34.29% (12/35)	37.04% (10/27)	0.822

Data are presented as the mean ± standard deviation for normally distributed continuous variables and as numbers (percentages) for categorical variables. Statistical comparisons were performed using the independent-samples t-test for normally distributed continuous variables and the chi-square test or Fisher’s exact test for categorical variables, as appropriate

## Discussion

In this retrospective comparative study, the modified one-layer microsurgical VV achieved patency and pregnancy rates comparable to those of the formal two-layer technique, while significantly reducing the operative time and technical difficulty. Importantly, these benefits were achieved without an increase in postoperative complications or reobstruction. Taken together, these findings suggest that the modified one-layer technique may provide a simpler and clinically effective alternative to the formal two-layer VV.

Our findings are consistent with the available evidence comparing one-layer and two-layer VV. The Vasovasostomy Study Group reported no significant differences in postoperative patency and pregnancy rates between the two techniques [11], and a subsequent meta-analysis and systematic review by Lindsey et al. reached a similar conclusion [20]. Microsurgical reconstruction remains a highly effective and cost-efficient option for men with vasal obstruction who desire natural conception, particularly when compared with ART, which may impose substantial financial and treatment burdens and may be less suitable for couples pursuing multiple reproductive attempts [18, 21, 22]. Although robot-assisted VV has shown comparable or potentially favorable outcomes in small studies, its broader adoption remains constrained by cost, availability, and training requirements [23, 24]. Consequently, refinement of conventional microsurgical VV remains clinically relevant.

The technical rationale underlying our modification may explain the observed outcomes. The formal two-layer microdot technique enables precise mucosal approximation, even when a substantial luminal diameter discrepancy exists between the two vasal ends [15, 17, 24]. However, placement of multiple mucosal sutures is technically demanding and may prolong operative time [18]. Moreover, extramucosal knots may induce tissue reactions, thereby promoting fibrosis and increasing the risk of anastomotic stenosis [13, 25]. By contrast, the conventional one-layer approach is less technically demanding and requires fewer sutures, potentially reducing the risks of suture granuloma formation and subsequent anastomotic stricture [26]. Nevertheless, prolonged obstruction often results in a marked diameter discrepancy between the testicular end and the abdominal end of the vas deferens. Under these circumstances, the conventional one-layer technique may result in uneven suture distribution, imprecise mucosal apposition, and inadequate anastomotic sealing, thereby increasing the risks of anastomotic leakage, sperm granuloma formation, luminal stenosis, and recurrent obstruction. Previous attempts to simplify VV, including the use of limited full-thickness sutures combined with biomaterial wrapping, have shortened operative time [4, 27–31], however, the efficacy and safety of these adjunctive biomaterials remain to be rigorously validated [31].

Our modified one-layer technique was designed to preserve the accuracy of the microdot method while simplifying suture placement. Six microdot-guided full-thickness sutures facilitate precise alignment and mucosal apposition. Six additional seromuscular sutures are placed in a staggered pattern to cover the intervals between the full-thickness sutures, thereby reinforcing the anastomosis and improving watertightness. This configuration combines the principal advantages of the formal two-layer and conventional one-layer techniques: accurate mucosal approximation and secure sealing with reduced suturing complexity and operative time.

The comparable patency and pregnancy rates between the two groups are consistent with previous studies demonstrating that one-layer and two-layer VV can achieve similar reproductive outcomes [1, 20, 24]. The shorter operative time associated with the modified technique is clinically meaningful, as microsurgical VV involves prolonged, highly precise suturing and places considerable technical, physical, and psychological demands on surgeons [1, 13, 14, 18]. By simplifying full-thickness suture placement while preserving a tension-free, watertight anastomosis and avoiding knots within the muscularis, this technique may enhance procedural efficiency without compromising anastomotic quality. Moreover, the less steep learning curve may facilitate adoption by novice microsurgeons. Therefore, proficiency in this modified one-layer technique may provide a practical alternative for many surgeons and potentially expand access to effective microsurgical reconstruction. A notable strength of the present study is the relatively homogeneous study population, in which all patients underwent vasectomy reversal. This minimized potential confounding caused by different etiologies of vas deferens obstruction, such as herniorrhaphy-related injury or previous reconstructive surgery, thereby strengthening the reliability of the comparison.

## Limitations of the study

This study has several limitations. First, its retrospective design and limited sample size may introduce selection bias and constrain the generalizability of the findings. Second, the relatively short median follow-up duration precluded a robust evaluation of long-term reobstruction. Large, prospective randomized controlled studies with longer follow-up are warranted to further validate these findings and evaluate long-term durability.

## Conclusions

Compared with formal two-layer microsurgical VV, the modified one-layer technique achieved comparable patency and pregnancy rates while reducing technical difficulty, shortening operative time, and facilitating skill acquisition. It combines the advantages of the conventional one-layer and formal two-layer techniques, and may therefore represent an effective microsurgical approach to VV; however, further studies are needed to confirm its superiority.

## Data Availability

No datasets were generated or analysed during the current study.

## Abbreviations

ART: Assisted reproductive technology
VE: Vasoepididymostomy
VV: Vasovasostomy
